# The Antibiotic Drug Tigecycline: A Focus on its Promising Anticancer Properties

**DOI:** 10.3389/fphar.2016.00473

**Published:** 2016-12-02

**Authors:** Zhijie Xu, Yuanliang Yan, Zhi Li, Long Qian, Zhicheng Gong

**Affiliations:** ^1^Department of Pathology, Xiangya Hospital, Central South UniversityChangsha, China; ^2^Department of Pathology, School of Basic Medicine, Central South UniversityChangsha, China; ^3^Department of Pharmacy, Xiangya Hospital, Central South UniversityChangsha, China; ^4^Institute of Hospital Pharmacy, Central South UniversityChangsha, China; ^5^Center for Molecular Medicine, Xiangya Hospital, Key Laboratory of Molecular Radiation Oncology of Hunan Province, Central South UniversityChangsha, China

**Keywords:** tigecycline, anti-cancer effects, mitochondrial function, Akt signaling, side effects, clinical trials

## Abstract

Tigecycline (TIG), the first member of glycylcycline bacteriostatic agents, has been approved to treat complicated infections in the clinic because of its expanded-spectrum antibiotic potential. Recently, an increasing number of studies have emphasized the anti-tumor effects of TIG. The inhibitory effects of TIG on cancer depend on several activating signaling pathways and abnormal mitochondrial function in cancer cells. The aim of this review is to summarize the cumulative anti-tumor evidence supporting TIG activity against different cancer types, including acute myeloid leukemia (AML), glioma, non-small cell lung cancer (NSCLC), among others. In addition, the efficacy and side effects of TIG in cancer patients are summarized in detail. Future clinical trials are also to be discussed that will evaluate the security and validate the underlying the tumor-killing properties of TIG.

## Introduction

Advances in genomic knowledge are providing attractive insights into the biology of human cancers, but the development of novel pharmacologic agents has not proceeded as quickly (Waldron, 2016). Identification of novel molecular events, such as gene expression signatures and mutation assessment, has yielded tremendous insights into cancer pathogenesis. However, many molecular expression patterns, highlighted by studies over the past decade, such as loss- or gain-of-function mutations in the WIP1 (wild-type p53-induced phosphatase 1) gene, have not produced straightforward therapeutic targets (Ruark et al., 2013). An alternative strategy for therapeutic identification is to screen on- and off-patent drugs with potential anti-cancer properties that are not directly related to the gene mutations.

Tigecycline (TIG), an FDA (U.S. Food and Drug Administration) approved glycylcycline antimicrobial agent, is widely used to treat complicated intra-abdominal infections (IAIs) and skin-structure infections (SSIs) (Bradford et al., 2005). Studies have indicated that TIG exhibits broad spectrum anti-bacterial ability against Gram-positive and Gram-negative bacteria, including *Staphylococcus aureus* (Sader et al., 2016), *Acinetobacter baumannii* (Rao et al., 2016), Enterobacteriaceae (Thaden et al., 2016), among others. As an expanded-spectrum antibiotic, TIG is clinically available for bacterial eradication with a good safety and tolerability profile, particularly for cancer patients (Lauf et al., 2014). Because of intensive myelosuppressive chemotherapy (Razzouk et al., 2006) and surgical site infections, cancer patients are susceptible to infections (Teillant et al., 2015). In a multicenter, open-label, randomized and superiority trial, 390 febrile neutropenic participants with cancer were enrolled and randomly assigned to receive piperacillin/tazobactam with or without TIG. Compared with the monotherapy group, the clinical outcome is significantly higher in the combination group, indicating that TIG could be considered as one of the first-line empiric antibiotic therapies for cancer patients with a pathogen infection (Bucaneve et al., 2014). In addition, a growing body of evidence shows that TIG possesses direct anti-tumor effects *in vivo* and *in vitro*. Skrtic’s group found that TIG could selectively induce cell death in a panel of leukemia cell lines without obvious side effects on normal hematopoietic cells. Meanwhile, combining TIG with daunorubicin or cytarabine, two standard chemotherapeutics that are used for acute myeloid leukemia (AML), exhibits an additive or synergistic cytotoxic effect (Jaras and Ebert, 2011; Schimmer and Skrtic, 2012). Therefore, in this review, we mainly focus on updating the findings regarding the anti-tumor activity of TIG and discuss well-investigated signaling molecules related to the anti-cancer effects of TIG. In addition, as a promising chemotherapy drug which may be used clinically in the future, the effectiveness and adverse effects of TIG will also be discussed.

## Mitochondrion as a Target of Tig

Mitochondria, the energy house of cells, are semiautonomous organelles, as they contain their own genetic material – mitochondrial DNA (mtDNA). The mtDNA is a double-stranded circular genome without introns that can be transcribed into 2 ribosomal RNAs (rRNAs) and 22 transfer RNAs (tRNAs) and encodes 13 of 90 proteins required for electron transport by the respiratory chain. In addition, mitochondrial protein synthesis depends on the unique protein translation mechanism, including particular initiation and elongation factors that differ from eukaryotic cytosolic factors (Zong et al., 2016). Multiple studies have illustrated that mitochondria are not only critical for normal cell function, they also play a role in malignant tumor progression (Quiros et al., 2015; Bender and Martinou, 2016).

Since they were discovered in approximately the 1890s by Richard Altmann and Carl Benda, two famous German scientists, mitochondria have attracted increasing interest of scientists. In the following decades, the understanding of mitochondrial function has grown enormously thanks to advances in biochemical and genetics technologies (Picard et al., 2016). In physiological conditions, a major function of mitochondria is to generate the energy-rich molecule adenosine triphosphate (ATP), promoting cell survival. Moreover, mitochondria are signaling structures that allow the cell to adapt to the environment by sensing stress. Many other biologic behaviors support these mitochondrial functions, including biosynthetic metabolism, fusion dynamics, oxidative stress responses, and so on (Nicholls and Budd, 2000). Recent studies have highlighted the importance of the mitochondrial machinery in human diseases, such as age-related disorders (Sun et al., 2016) and cancers (Vyas et al., 2016). Numerous studies on the mitochondrial roles in cancer have initiated a novel frontier focus, suggesting the multifaceted functions of mitochondria in tumorigenesis and progression. In some cancer types, owing to mitochondrial functional defects, malignant cells undergo aerobic glycolysis to maintain rapid proliferation and resistance to therapeutic stress, which is known as the Warburg effect (Flaveny et al., 2015). Compared with normal cells, cancer cells exhibit markedly upregulated glucose uptake and aerobic glycolysis, increasing the yield of biosynthetic intermediates, providing essential anabolic molecules for cell proliferation and tumor growth (Ribas et al., 2016). Gius’s study suggested that knockdown of sirtuins, a cellular energy sensor, could increase glycolytic metabolism, further promoting mammary tumor growth (Park et al., 2016). Loss of mitochondrial PTEN-induced kinase 1 (PINK1) could promote glioblastoma growth by increasing the Warburg effect *in vitro* and *in vivo* (Agnihotri et al., 2016). However, a more complex picture is emerging in which some cancer patients depend on the mitochondria respiratory function. Supporting this concept, Alam et al. (2016a) demonstrated that enhanced mitochondrial aerobic respiration is necessary for many types of cancer cells to gain tumorigenic and drug-resistant potential, such as non-small cell lung cancer (NSCLC) and breast cancer. Inhibition of mitochondrial respiration by Hedgehog inhibitors, such as cyclopamine tartrate, could strongly interfere with cell proliferation and induce apoptosis in NSCLC (Alam et al., 2016b). In addition, studies have demonstrated that based on the context, the mitochondrial mass can serve as either a pro-survival or pro-death modulator in tumor development and progression (Joshi et al., 2016). These “pleiotropic effects” could be influenced by genetic, environmental and tissue-derived differences between tumors.

**FIGURE 1:**
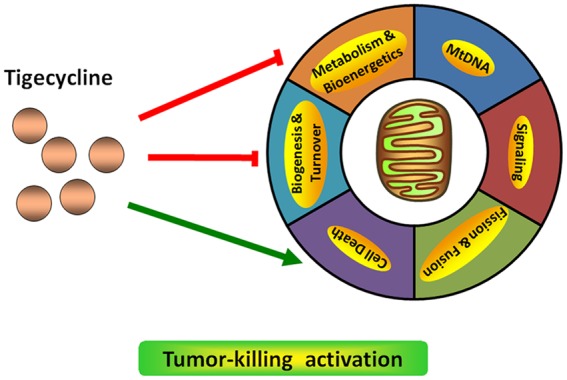
**The effect of the antibiotic drug TIG on mitochondrial function in cancer**.

Targeting mitochondrial functions, such as mitochondrial biogenesis, is a successful strategy for cancer therapeutics (**Figure 1**). Skrtić et al. (2011) found that TIG has selective toxicity on leukemia cells, especially leukemia stem and progenitor cells *in vitro* and *in vivo*, and this cell cytotoxicity of TIG depends on the intact respiratory chain. However, TIG-resistant cancer cells show more resistance to hypoxia with an upregulated hypoxia-inducible factor 1α (HIF-1α) level from defective oxidative phosphorylation (Jhas et al., 2013). Using the haplo-insufficiency profiling screen, a well-validated, automated and high-throughput chemogenomic assay platform developed in yeast, mitochondrial protein synthesis has been identified as the mechanism of TIG-induced lethality. Knockout of mitochondrial elongation factor Tu (mEF-Tu), a key modulator involved in mitochondrial protein translation, could significantly reproduce the anti-leukemia potential of TIG (Skrtić et al., 2011). The same group also developed a new formulation that could enhance the stability of TIG in saline solution as well as preserve the agent’s anti-leukemic activity. The elements added to this formulation are mainly ascorbic acid and pyruvate (Jitkova et al., 2014). Apart from its anti-leukemic effect, recent studies have demonstrated that TIG could target multiple cancers by impairing mitochondrial functions (Lamb et al., 2015). For example, Jia et al. (2016) found that TIG could significantly reduce growth and induce apoptosis in various NSCLC cell lines through inhibition of mitochondrial function. Inhibiting the mitochondrial gene expression and translation pathway by TIG could induce MYC oncogene-dependent tumor cell death, including the osteosarcomas (Oran et al., 2016) and lymphomas (D’Andrea et al., 2016). As the mitochondrial energy metabolism provides distinct pro-survival benefits to diffuse large B-cell lymphomas (DLBCLs), pharmacological perturbation of the mitochondrial translation pathway with TIG is proved to be selectively toxic to DLBCL cell lines (Norberg et al., 2016). In addition, another group has identified that TIG could serve as a potential new therapeutic drug for treatment of retinoblastoma (RB1) -deficient breast cancer (Jones et al., 2016). Therefore, an important consideration in anti-cancer fields will be addressing mitochondrial signaling modulation with chemical compounds.

## Akt Signaling as a Target of Tig

Akt, also known as protein kinase B (PKB), belongs to an evolutionarily conserved serine-protein kinase family and participates in cell homeostasis maintenance by regulating numerous downstream effectors. The Akt family mainly contains three members, Akt1 (PKBα), Akt2 (PKBβ), and Akt3 (PKBγ), which share more than 80% structure homology. All of these isoforms contain three similar domains with an N-terminal pleckstrin homology motif, central kinase catalytic motif, and C-terminal hydrophobic motif tail (Sussman et al., 2011). Since its discovery, numerous efforts have been made to clarify the mechanism of Akt activation. Studies have indicated that the Akt signaling pathway could be activated by receptor tyrosine kinases (RTKs), including insulin-like growth factor 1 receptor (IGF-1R), epidermal growth factor receptor (EGFR) and others (Manning and Cantley, 2007). Upon stimulation by RTKs, phosphatidylinositol 3-kinase (PI3K) is recruited to the plasma membrane, which subsequently catalyzes the phosphorylation of phosphatidylinositol 4,5-bisphosphate (PIP2) to generate phosphatidylinositol 3,4,5-triphosphate (PIP3). Then, the accumulation of PIP3 activates Akt by phosphorylation modification at two regulatory sites, Thr308 and Ser473 (Cantley, 2002). In addition to RTKs, Akt activity can be modulated by lipid and protein phosphatases, such as phosphatase and tensin homolog (PTEN) and protein phosphatase 2A (PP2A). PTEN is a negative modulator of the Akt signaling pathway, and inhibition of PTEN by a small-molecule inhibitor could significantly activate Akt (Shojaee et al., 2016). Similarly, PP2A can down-regulate Akt activation by directly promoting Thr308 dephosphorylation (Liu et al., 2016). Additionally, once Akt is locked in the active confirmation, it can regulate more than 100 down-stream factors that are involved in diverse cellular functions, including cell proliferation, apoptosis, metabolism, and so on. These direct substrates of Akt all share a consensus phosphorylated minimal motif (R-X-R-X-X-pS/pT) (Carmona et al., 2016). For example, AKT phosphorylates glycogen synthase kinase-3β (GSK3β) at Ser9 and thus inactivates it, which promotes Erb-B2 receptor tyrosine kinase 2 (ErbB2)-mediated cardiomyocyte proliferation (D’Uva et al., 2015). Akt-dependent phosphorylation of mTOR complex 1 (mTORC1) plays an important role in the self-renewal capacity of hematopoietic or leukemia stem cells (Lechman et al., 2016).

To date, molecular screening of human disease models, particularly for cancer, has identified a series of alterations that affect the Akt signaling pathways. It is of particular interest to explore the possible molecular mechanism underlying Akt activation, which is a potential contributor to cancer cell development (Fortin and Mak, 2016). The most common type of mechanism is from loss- or gain-of-function. Due to frequent mutation, loss-of-function of PTEN is a canonical event in cancer cells. PTEN loss results in continuous activation of Akt signaling, which is correlated with the shorter overall survival of BRAF^V 600E^ mutated melanoma patients (Bucheit et al., 2014). However, using whole-genome sequencing, Costa et al. (2015) identified a novel point mutation (A126G) in the PTEN protein. They found that unlike canonical loss-of-function mutants, A126G mutation could produce an enzymatic gain-of-function in PTEN, activating the Akt signaling pathway and prompting cell proliferation in prostate cancer (Costa et al., 2015). These findings suggest the crucial role of the PTEN mutation in the Akt pathway in cancer. The second mechanism is a compensatory effect. Inhibition of Akt has a negative feedback effect on RTK activation, which then enhances Akt activation. Akt inhibitors, AKTi-1/2 and MK-2206, could re-activate RTK signaling in a FOXO-dependent manner, attenuating the anti-tumor effects of these inhibitors (Chandarlapaty et al., 2011). As a result, it is critically important to clarify the detailed mechanism of the Akt signaling pathway, which will help develop a more efficient treatment strategy, including pharmacologic therapies.

**FIGURE 2:**
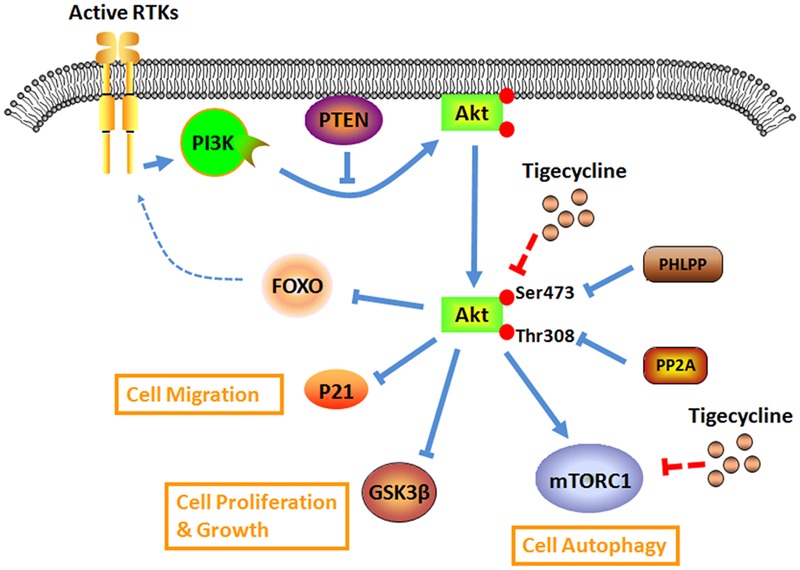
**Overview of the antibiotic drug TIG on the aberrant Akt signaling for cancer research and therapy**.

Antibiotic TIG has an attractive anti-proliferation effect on neuroblastoma cells by dephosphorylating Akt and its down-stream targets *in vitro* and *in vivo* (**Figure 2**). The Akt activator IGF-1 significantly rescues the inhibition effects of TIG (Zhong et al., 2016). Further study demonstrated that after treating glioma U87 and U128 cells with TIG, the miRNA-199b-5p level obviously increased and the level of HES family BHLH transcription factor 1 (HES1), a target of miRNA-199b-5p, obviously decreased. Moreover, TIG decreased Akt phosphorylation at Ser473 and increased its target p21 level via the miRNA-199b-5p-HES1 axis (Yang et al., 2016). Another study showed that TIG could induce cell G1/S phase arrest and suppress migration/invasion by down-regulating the level of p21 in melanoma A375 and MV3 cell lines (Hu et al., 2016). These contrary roles of p21 might be due to its subcellular localization. In its traditional function, nuclear p21 acts as a dominant cyclin-dependent kinase (CDK) inhibitor that facilitates tumor suppression. While in the cytoplasm, p21 also exhibits oncogenic properties by interacting with a large set of molecules involved in cell proliferation, apoptosis, metastasis, and so on (Abbas and Dutta, 2009). In addition, it confirmed that the mammalian target of rapamycin (mTOR) also serves as a central factor underlying the Akt signaling pathway. Cui’s group found that TIG could induce autophagic cell death in gastric cancer cells, GAM-016 and MKN-45, by abrogating mTOR phosphorylation at the Ser2448 position (Tang et al., 2014). Taken together, TIG could act as a powerful candidate for intervening in the Akt signaling pathway for cancer therapy.

## Wnt/β-Catenin Signaling as a Target of Tig

Wnt, a secreted and lipid-modified glycoprotein, can activate a variety of cellular surface receptor-mediated signal transduction pathways that are involved in embryonic development and normal tissue homeostasis. In the canonical Wnt pathway, Wnt signaling inhibits β-catenin degradation, which can result in changes in various genes at the transcription level, such as the transcription factors HOXA5 (Ordonez-Moran et al., 2015) and ZEB1 (Wang et al., 2014). Several studies have revealed that activation of the Wnt signaling pathway is a hallmark of many human cancers (Clevers, 2006). Therefore, identification of the components of the Wnt/β-catenin pathway can provide promising targets for developing therapeutic agents. In a recent report, using the TOPflash reporter activity assay, Li et al. (2015) found that TIG decreased the Wnt/β-catenin-mediated transcription in cervical squamous cell carcinoma and that overexpression of exogenous β-catenin could abolish the inhibitory effects of TIG on cancer cells.

## Safety and Efficacy

TIG is a novel injectable antimicrobial with a broader spectrum of *in vivo* activity against a large number of Gram-positive as well as Gram-negative pathogenic bacteria. Some studies so far have evaluated its use in different hospital settings and microorganisms (**Table 1**). A recent retrospective study in patients with nosocomial IAIs showed that clinical response rate with TIG at standard dosage (initial dose of 100 mg, followed by 50 mg every 12 h) is approximately 78.3%., and none of the patients discontinued TIG treatment due to the side effects (Maseda et al., 2013). Furthermore, higher dosage TIG regimens (initial dose of 200 mg, followed by 100 mg every 12 h) could be potentially used to treat the severe hospital-acquired infections (Ramirez et al., 2013). In addition, three pediatric cases all demonstrated that TIG could effectively kill the multidrug-resistant pathogens in children with AML, with favorable safety and toleration (Dinleyici et al., 2010; Ozdemir et al., 2012; Tugcu et al., 2015). Nevertheless, for patients who have been long-term exposed to broad-spectrum antibiotic TIG, conditional pathogens such as *Chryseobacterium indologenes* could cause various types of refractory infectious events (Chen et al., 2013). Furthermore, according to the FDA drug safety communication, researchers pointed out that compared to comparator-treated patients, the TIG-treated patients showed an increased risk of mortality ranging from 3 to 4%, with the clinical cure rates increased from 78.5 to 81.3% (Gardiner et al., 2010; Averbuch et al., 2013). Moreover, data of 35 febrile neutropenic patients with hematological malignancies indicated that 2.9% of patients treated with TIG have to be terminated owing the intolerable nausea and headache (Schwab et al., 2014). Recently, Maximova et al. (2013) reported the myelotoxicity of TIG in two boys who underwent the bone marrow transplantation. Through analyzing the clinical characteristics, they found that TIG could significantly reduce the cellular viability of bone marrow cells in a dose-dependent manner (Maximova et al., 2013). Another case of the toxicity of TIG has been reported by Mcgovern et al. (2014) via analysing the subject data from phases III and IV comparative studies, which demonstrated adverse events of pancreatitis are also uncommon in patients treated with TIG, with an occurrence of <1% (McGovern et al., 2014). These occasional serious adverse reactions of TIG should arouse urgent attention of the researchers and clinicians, which requires further evaluation.

**Table 1:** The efficacy and side-effects of TIG in clinical cases.

Patients	Diseases	Response rate	Side-effects	Reference
215	*Chryseobacterium indologenes* infections	51.9% for blood isolates; 39.1% for sputum isolates	N/A	Chen et al., 2013
110	Cancer	64%	Gastrointestinal disorders	Chemaly et al., 2009
199	Intra-abdominal infections	86.5% for ME populations, 81.7% for mITT populations	Gastrointestinal disorders	Chen et al., 2010
24	Cancer	48%	4% increased liver enzyme serum concentrations	Secondo et al., 2010
2	BMT	N/A	Marrow toxicity	Maximova et al., 2013
114	Hospital-acquired pneumonia	85% for TIG 100 mg; 69.6% for TIG 75 mg	Gastrointestinal disorders	Ramirez et al., 2013
23	Intra-abdominal infections	78.3%	None	Maseda et al., 2013
170	Secondary bacteremia	81.3%	2.2% mortality	Gardiner et al., 2010
35	Hematological malignancies	43%	Gastrointestinal disorders, Liver and renal toxicity	Schwab et al., 2014
3788	Inflammation	N/A	<1% pancreatitis	McGovern et al., 2014
1	AML	N/A	None	Dinleyici et al., 2010

*N/A, not available; ME, microbiologically evaluable patients; mITT, modified intent-to-treat patients; BMT, bone marrow transplantation; AML, acute lymphoblastic leukemia.*

Apart from the infrequent complications mentioned above, the most common adverse effects (incidence >5%) associated with the use of TIG have been confined to gastrointestinal symptoms, including nausea, vomiting, and diarrhea in the infected population, particularly in patients with cancers (Kosmidis and Chandrasekar, 2012). Phase III clinical trials conducted by Cooper’s group suggested that upon treated by TIG, the incidence of nausea, vomiting and diarrhea are 21.6, 12.4, and 5.2%, respectively (Chen et al., 2010). Another retrospective review of 110 cancer patients reported that about 64% participants have an overall clinical response to TIG. Interestingly, the incidence of gastrointestinal symptoms dropped below 5% (5% for mile nausea, 2% for vomiting, and 4% for diarrhea, respectively), which are mainly due to the antiemetics and ventilator support at the start of TIG therapy (Chemaly et al., 2009). These findings suggest that administration of supportive medications prior to TIG could significantly reduce the incidence of gastrointestinal side-effects. Finally, other mild side-effects associated with TIG administration were also recorded, such as increased serum concentrations of liver enzyme (Secondo et al., 2010) and liver and renal toxicity (Schwab et al., 2014). Collectively, these studies illustrate the unique clinical presentation in the process of treatment with TIG for infections in patients, particularly in cancer patients. Evidently, more investigations on clinical application of TIG are required to reveal the details regarding the safety and efficacy of TIG. Without any doubt, these observations will shed more light on how to treat infection or cancer with TIG properly without inducing apparent and serious adverse effects.

## Perspective in Clinical Trials

Up to now, several therapeutic molecules that inhibit cancer-associated signaling pathways, such as dichloroacetate (DCA), an inhibitor of the mitochondrial pyruvate dehydrogenase kinase, have been used in clinical trials^1^. As Akt signaling and mitochondrial biology both participate in the physiological behaviors, the clinical use of their corresponding inhibitors would cause side effects in a certain extent. Chu et al. (2015) conducted an open-labeled, single-arm, dose-escalation study of DCA in 24 patients with advanced solid malignancies and found that the response to DCA with grade I-II toxicities can be evaluated in most patients. Another dose/schedule-finding study by Tolcher et al. (2015) indicated that patients with advanced treatment-refractory solid tumors show partial responses to MK-2206 treatment with obvious dose-limiting toxicity. As a result, future clinical trials to verify the efficacy of new anti-cancer compounds in multiple human cancers are now clearly warranted. In a clinical trial conducted by Bucaneve et al. (2014), the combination of piperacillin/tazobactam and TIG was more effective for treating hematologic cancer patients (Bucaneve et al., 2014). Moreover, TIG is well tolerated and relatively safe, apart from mild gastrointestinal adverse events (Garrison et al., 2005). Recently, based on the pre-clinical data mentioned above, TIG has recently completed a Phase I clinical trial for the treatment of AML with a favorable safety profile at doses 300 mg/day (clinicaltrials. Gov ID: NCT01332786) (Reed et al., 2016). Although only a few issues about the direct tumor-killing effect of TIG have been preliminarily evaluated in clinical trials so far, studies on the great biological and clinical efficacy of TIG will light up a promising way in cancer treatment.

## Conclusion

In recent decades, molecular screening of cancer tissues and cells by high-throughput bioinformatics platforms is starting to guide the target choice for therapeutic interference. Investigation of the genetic and biological diversity between normal and cancer cells would help to develop more effective tumor-killing agents without obvious side effects. For instance, with its selectively potential for malignant cells, TIG could serve as a lead candidate for novel chemotherapy- cytotoxic drug development. In mechanism analysis, the combination of a small compound screen, yeast chemogenomic platform and further *in vitro* and *in vivo* experiments is conducive to identifying dysregulation signaling as the target for candidate compounds, such as TIG. Furthermore, given the issues with clinical application, future studies should focus on the combined effects between TIG and standard chemotherapy drugs to effectively treat cancer patients.

## Author Contributions

ZX designed the work. ZX, YY, and LQ wrote the manuscript. ZL and ZG revised the manuscript. All authors reviewed and approved the final version of the manuscript.

## Conflict of Interest Statement

The authors declare that the research was conducted in the absence of any commercial or financial relationships that could be construed as a potential conflict of interest.

## Footnotes

1 https://clinicaltrials.gov/
